# Control of neglected tropical diseases in Asia Pacific: implications for health information priorities

**DOI:** 10.1186/2049-9957-1-3

**Published:** 2012-10-25

**Authors:** Robert Bergquist, Maxine Whittaker

**Affiliations:** 1 Ingerod, 454 94, Brastad, Sweden; 2 Australian Centre for International and Tropical Health, School of Population Health, University of Queensland, Herston, Qld, 4006, Australia

**Keywords:** Neglected tropical diseases, NTD, Health information systems, Poverty, Disease surveillance, Control programmes, Asia Pacific

## Abstract

Poverty magnifies limitations posed by traditional biases and environmental risks. Any approach towards disease control needs to recognise that socially embedded vulnerabilities can be as powerful as externally imposed infections. Asia Pacific has a specific panorama of infectious diseases, which, in common with other endemic areas, have a tendency to emerge or re-emerge if not carefully monitored. Sustained control aiming at elimination requires strong emphasis on surveillance and response. Well-designed informatics platforms can improve support systems and strengthen control activities, as they rapidly locate high-risk areas and provide detailed, up-to-date information on the performance of ongoing control programmes.

## Multilingual abstracts

Please see Additional file 1 for translations of the abstract into the six official working languages of the United Nations.

## Background

**Figure 1:**
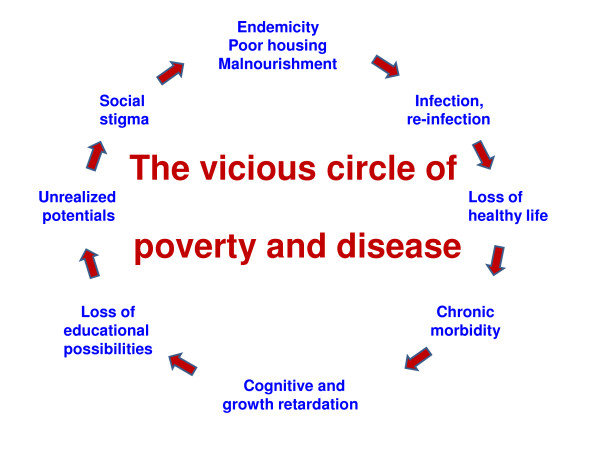


The common denominator for being at risk for neglected tropical diseases (NTDs) is poverty, a condition that makes people vulnerable to them due to environmental risks as well as social vulnerabilities varying from gender bias to substance abuse or harmful feeding habits. These vulnerabilities sustain the problem as they are widespread and the forces upholding them are as much shaped by the economy and society as by the environment. Over two billion people in the world are affected by the group of infections known as the NTDs [1]; tropical because of their spatial distribution and neglected because they do not receive sufficient interest and research funding. Not surprisingly, the NTDs are common in areas where the human development index (HDI) is low. They are in truth neglected people’s neglected diseases since they, beyond their direct impact on health, feed the vicious cycle of poverty and disease (Figure 1) that leaves children unable to go to school and adults incabable of working or fully participating in community life [2]. 

Two of the United Nation’s eight millennium development goals (MDGs), agreed by member states as critical for the world to continue to develop on a global scale [3], are related to the area of public health. One concerns child mortality and maternal health and the other communicable diseases. Research on aspects of these two MDGs is progressing well, but the various infectious diseases affecting marginalized people in the less developed countries (LDCs) are still far from under control. Indeed, 12 years on, the NTDs still top the list of health crises in the developing world. The combined burden of soil-transmitted helminthiases (STHs), snail-borne trematode infections, and vector-borne infections in the endemic countries presents a formidable challenge for the national Ministries of Health due to insufficient data and lack of resources. The progress achieved in reaching populations in need of treatment for lymphatic filariasis (LF), schistosomiasis and STHs notwithstanding, most of the NTDs are generally not controlled at all, and those affected by them lack adequate assistance. The seriousness is compounded by the fact that many NTDs are chronic, not easily cured, and as a rule lead to long-term incapacity and suffering. The predicament is particularly grave in the region commonly referred to as Asia Pacific even if the human development index (HDI) is relatively high in parts of the region. In fact, the burden of infectious diseases in Asia Pacific is second only to that of sub-Saharan Africa. Although it is often said that the great majority of the NTDs can be prevented or eliminated with inexpensive or donated medicines, there are exceptions. There is no safe and effective treatment for leishmaniasis, and dengue stands out as the most rapidly spreading vector-borne disease in the world with Asia Pacific harbouring 75% of all cases [4]. The threat of dengue is so much more than an alert among others as there is neither a vaccine nor a drug available to prevent or cure this infection.

Research on resource allocation and planning for approaches addressing priority gaps has a big role to fill here and direct collaboration with health staff in the endemic countries together with support for the development of health information systems (HIS) are vital. Improved national HIS institutions are the key to better public health management, which should not only include collection and compilation of data but also deal with analysis, surveillance, follow-up and, especially, dissemination of information and health planning at the national level.

## Commentary

One explanation for the slow awakening to the role of NTDs is that their mortality is generally low and they often present with only subtle symptoms that hamper assessment of their burden. For example, hookworm infection and schistosomiasis are leading causes of anaemia [5,6] that can result in delayed cognitive development, poor school/work performance and impaired growth [7-9]. In addition, lack of sufficiently sensitive diagnostic tools undermine reliable assessments of prevalence and intensity of disease [10,11], and this weakness becomes acute in areas where disease control has progressed significantly since the combination of low levels of infection and low prevalence produces a negative bias. Ignorance of the real situation leads to faulty impact estimates, sometimes leading to redirected control activities and re-emergence of disease. All this conspires to keeping us in the dark with respect to chronic afflictions, which only become manifest long after infection.

To some extent, the pathological ramifications induced by the NTDs remain unknown since there are few studies on how concurrent infections influence each other. Not only do morphological similarities between some parasites (and/or their eggs) blur the diagnostic picture, the dearth of representative samples due to interactions between parasite species adds to the difficulties in assessing the impact [12,13]. Frequency of infection and specific characteristics of multi-parasitism are seldom available in routinely collected data and this leads to ill-defined morbidity assessments. For example, the spatial overlap between tuberculosis (TB), malaria and infections by the human immunodeficiency virus (HIV) on the one hand, and the NTDs on the other, marginalizes the impact of the latter group [14]. In addition, the influence of animal reservoirs on the epidemiological profile of diseases such as the food-borne trematode infections (FBT) and the cestode infections is often overlooked, but general evidence provided by the veterinary public health sector suggests that the human dimension of many zoonotic infections is strongly underestimated.

The goal of HIS is to produce quality and timely information for evidenced-based decisions and interventions. However, HIS is generally weak in the developing world since donors tend to rely more on national surveys and also still favour a vertical approach [15], while the key for sustained progress is to enhance the entire system rather than focus on individual diseases. However, the MDGs have ushered in an unprecedented demand for health information, while current administrative decentralization has led to increased reporting requirements and performance-based resource allocation. Since critical analysis and assessment of appropriate policies and strategies need a well-functioning HIS department, strengthening of human resources and coordination/integration of data collection is essential. Support is particularly important as the developing countries generally lack public health capability and are not equipped to deal with the many problems threatening their progress. Stimulation of policy research facilitating the control of diseases of regional importance is a question of overriding importance in convincing national governments to strengthen their own HIS capabilities and to better orientate their control programmes towards public health activities that can be sustained. To gain the upper hand in controlling the NTDs, the tools of recognition and discovery such as diagnostics, geospatial sciences and health metrics should be emphasized. This approach must go hand in hand with strengthening surveillance and the establishment of an early warning system (EWS) approach with respect to infectious diseases [16,17]. However, progress needs also to be linked to cross-cutting themes such as socioeconomic issues and matters of more general impact, for example, climate change. From the human resource point of view, training and broadening of capacity should be strengthened accordingly.

Some diseases are becoming eliminated in parts of the world, but the risk is that they re-emerge if not carefully monitored. Notably, China plans to eliminate schistosomiasis and malaria by 2020. This should produce a substantial increase in human welfare, underpin socio-economic growth and create a virtuous circle leading away from vulnerability. However, sustained success requires strong emphasis on surveillance. An approach utilizing well-designed informatics platforms has the potential to improve support systems and strengthen control activities as these can rapidly locate high-risk areas and retrieve all important data needed as well as provide detailed, up-to-date information on the performance of any ongoing control programme. Interestingly, the application of geographical information systems (GIS) and remote sensing from satellites are transforming epidemiological thinking into research agendas that offer solutions leading to the development of integrated systems for disease control and surveillance [18]. HIS has an important role to play in the development and implementation of surveillance and prediction of risk areas. In this connection, computerized informatics platforms based on geospatial technology should be considered. Indeed, open-source software platforms have been developed and used for risk assessment for two NTDs: dengue in Argentina [19] and schistosomiasis in China [20]. The former includes environmental, viral, entomological and social information for about 3,000 cities in that country. It updates regularly risk vectors for each locality according to the European Space Agency standards for space informatics and provides also specific maps modelling the dengue risk inside selected cities. The Chinese approach, focused on schistosomiasis risk, was developed by combining spatial data from Google Earth software with a GIS package, bundling the separate modules together with an Internet connection into a well-functioning ‘WebGIS’ system. These platforms are primarily aimed at becoming a dynamic tool for surveillance and response for health managers. The approach is very versatile in the sense that it provides evidence-based, near real-time disease status for many users simultaneously, thereby providing rapid information-sharing at all levels of decision-making and facilitating rapid point-of-care response.

## Conclusions

The vicious circle that connects poverty with infection and disease must be broken. Quality and timely information for evidenced-based decisions and interventions could provide a hiatus by supporting performance-based resource allocation leading to strengthening of the entire system rather than focusing on individual diseases. However, progress needs also to be linked to cross-cutting themes such as socioeconomic issues and matters of more general impact such as training and broadening of capacity. The geospatial sciences, health metrics and improved diagnostics have the potential to bolster recognition, discovery, surveillance and response.

## Abbreviations

FBT: Food-borne trematode infections; GIS: Geographical information systems; HDI: Human development index; HIV: Human immunodeficiency virus; HIS: Health information systems; LDCs: Less developed countries; LF: Lymphatic filariasis; MDGs: Millennium development goals; NTDs: Neglected tropical diseases; STHs: Soil-transmitted helminthiases; TB: Tuberculosis.

## Competing interests

The authors declare that they have no competing interests.

## Authors’ contributions

This work is the fruit of joint discussions between RB and MW over the last 4 months. RB drafted the original text. All authors read and approved the final manuscript.

## Authors’ information

RB holds a MD and a PhD from the Karolinska Institute, Stockholm, Sweden. He has spent his professional career in research on tropical diseases at the Karolinska Institute and the National Bacteriological Laboratory, Solna, Sweden. He is retired after 15 years with the UNICEF, UNDP, World Bank, WHO Special Programme for Research and Training in Tropical Diseases (TDR) and lives in Sweden.

MW is a professor of International and Tropical Health, Director of the Australian Centre for International and Tropical Health at the School of Population Health, University of Queensland in Herston, Australia. She is also the Executive Director of PacMISC and Co-Coordinator of the APMEN Secretariat.

## Supplementary Material

Additional file 1 Multilingual abstracts in the six official working languages of the United Nations. Click here for file
