# Supplementary material for: Control of neglected tropical diseases in Asia Pacific: implications for health information priorities
Source: Infect Dis Poverty. 2012 Oct 25;1:3. doi: 10.1186/2049-9957-1-3 (PMC3710194; doi:10.1186/2049-9957-1-3)
Supplement: Additional file 1 — Multilingual abstracts in the six official working languages of the United Nations. [file 2049-9957-1-3-S1.pdf]

Translation of the abstract into the six official working languages of the United Nations

الآثار المترتبة على أولويات المعلومات الصحية :"آسيا و المحيط الهادئ" السيطرة على الأمراض الاستوائية المهملة في

روبرت بيرجكويست، ماكسين ويتاكر

ملخص

إن أي منهج نحو مكافحة الأمراض يجب أن يدرك أن نقاط .يُعَظِم الفقر من القيود التي تفرضها التحيزات التقليدية و المخاطر البيئية آسيا المحيط الهادئ بها مشهد دائم .العدوى التي تفرضها العوامل الخارجية الضعف المضمنة اجتماعياً يمكن أن تكون بنفس قوة شأنها في ذلك شأن المناطق ,والذي لديه ميل للظهور أو معاودة الظهور لو لم يُرصد بعناية ,التغير و محدد للأمراض المعدية البرامج .لب تركيزاً على المراقبة و الاستجابةوالمراقبة الدائمة التي تهدف إلى القضاء على العدوى تتط .الموبوءة الأخرى حيث أنها تحدد سريعاً المناطق عالية الخطورة ,المعلوماتية جيدة التصميم يمكنها أن تحسن من أنظمة الدعم و تعزز أنشطة التحكم ,وتوفر معلومات حديثة وتفصيلية حول أداء برامج التحكم المستمر.

Translated from English version into Arabic by Berlant Tosson, through

TRANSLATORS WITHOUT BORDERS

亚太地区被忽视热带病的控制：卫生信息优先顺序的启示

Robert Bergquist, Maxine Whittaker

摘要

贫困扩大了传统偏见和环境风险所造成的局限性。在实施任何一种疾病控制的措施时需意识到社会内部的脆弱性与外加的感染一样危险。与其他流行地区一样，亚太区的传染病有其独特之处，如不细心监控，该地区的传染病有出现或再现的趋势。旨在消除疾病的持续防治需高度重视疾病监测和应对。精心设计的信息平台可以优化支持系统和加强防治活动，如快速确定高风险区域和提供防治项目实施效果的最新详细信息。

Translated from English version into Chinese by Yang Pin, through

CDC CHINESE CENTER FOR DISEASE CONTROL AND PREVENTION
NATIONAL INSTITUTE FOR PARASITIC DISEASES

Contrôle des maladies tropicales négligées en Asie-Pacifique : les conséquences sur les priorités en matière d'information sanitaire

Robert Bergquist, Maxine Whittaker

Résumé

La pauvreté amplifie les limites posées par les préjugés classiques et les risques environnementaux. Toute démarche en faveur du contrôle des maladies doit partir du principe que les vulnérabilités socialement ancrées peuvent être aussi dangereuses que les infections externes. La région Asie-Pacifique dispose d'une palette bien spécifique de maladies infectieuses qui ont tendance à apparaître ou à réapparaître si elles ne sont pas surveillées de très près – comme dans d'autres régions endémiques. Un contrôle soutenu et continu visant à éradiquer ces maladies nécessite que l'accent soit tout particulièrement mis sur la surveillance et la réponse. Des plateformes informatiques bien conçues peuvent permettre d'améliorer les systèmes de soutien et à renforcer les activités de contrôle ; en effet, elles localisent rapidement les zones à haut risque et fournissent des informations détaillées et actualisées sur la performance des programmes de contrôle en cours.

Translated from English version into French by Anne Diamantidis, through

TRANSLATORS WITHOUT BORDERS

Борьба с «забытыми» тропическими болезнями в странах Азиатско-Тихоокеанского региона: влияние на приоритеты в области информации по вопросам здравоохранения

Роберт Бергквист, Максин Уиттекер

Резюме

Нищета усиливает ограничения, создаваемые традиционными предрассудками и экологическими рисками. При любом подходе к вопросу борьбы с болезнями необходимо признавать, что социально закрепившиеся факторы уязвимости могут быть настолько же сильными, как и инфекции, навязанные извне. В Азиатско-Тихоокеанском регионе существует панорама инфекционных болезней, которые, как и в других эпидемиологических районах, имеют тенденцию повторного возникновения, если за ними не ведется тщательный надзор. Постоянная борьба с заболеваниями, нацеленная на их ликвидацию, требует концентрации усилий на вопросах отслеживания заболеваний и реагирования на них. Хорошо разработанные информационные платформы могут улучшить системы поддержки и усилить работу по борьбе с заболеваниями, так как они быстро определяют районы высокого риска и дают подробную, обновленную информацию о том, как идет выполнение существующих программ контроля.

Translated from English version into Russian by OWatts, through

TRANSLATORS
WITHOUT BORDERS

Control de las enfermedades tropicales desatendidas en la región de Asia-Pacífico: implicaciones para las prioridades de información sanitaria

Robert Bergquist, Maxine Whittaker

Resumen

La pobreza amplifica las limitaciones impuestas por los sesgos tradicionales y los riesgos medioambientales. Cualquier enfoque destinado al control de las enfermedades tiene que tener en cuenta que las vulnerabilidades socialmente enraizadas pueden ser tan poderosas como las infecciones impuestas desde el exterior. En la región de Asia-Pacífico existe un panorama específico de enfermedades infecciosas, las cuales, como ocurre en otras áreas endémicas, tienen tendencia a emerger o a reemerger si no son objeto de un meticuloso control. El control sostenido destinado a la erradicación requiere centrar un alto grado de atención en la vigilancia y la respuesta. Las plataformas informáticas bien diseñadas tienen la capacidad de mejorar los sistemas de apoyo y de reforzar las actividades de control, ya que localizan rápidamente las áreas de alto riesgo y proporcionan información detallada y actualizada sobre el rendimiento de los programas de control en marcha.

Translated from English version into Spanish by Elena de Teran Bleiberg, through

TRANSLATORS
WITHOUT BORDERS
